# Variations in Ecuadorian Cocoa Fermentation and Drying at Two Locations: Implications for Quality and Sensory

**DOI:** 10.3390/foods13010137

**Published:** 2023-12-30

**Authors:** Stefanie Streule, Susette Freimüller Leischtfeld, Martina Galler, Dominik Motzer, Monja Poulose-Züst, Susanne Miescher Schwenninger

**Affiliations:** 1 ZHAW Zurich University of Applied Sciences, Institute of Food and Beverage Innovation, Food Biotechnology Research Group, 8820 Wädenswil, Switzerland; stefanie.streule@zhaw.ch (S.S.); susette.freimueller@zhaw.ch (S.F.L.); dominikmotzer@hotmail.com (D.M.); poulose.monja@outlook.com (M.P.-Z.); 2 Lindt & Sprüngli, Seestrasse 204, 8802 Kilchberg, Switzerland; martinagaller@hotmail.com

**Keywords:** cacao nacional, cocoa bean fermentation, post-harvesting techniques, fermentation device, fermentation time, pre-drying, turning, sensory description

## Abstract

In Ecuador, various processes are applied during cocoa post-harvesting. This study, therefore, explored fermentation parameters across two locations with 2–7 independent runs, focusing on temperature, microbial counts, pH during fermentation and drying, and their impact on cocoa bean quality. Factors including fermentation devices (jute bags, plastic bags, and wooden boxes), pre-drying, turning during fermentation, fermentation duration, and drying temperature were investigated. Fermenting in plastic bags without pre-drying or turning and fermenting in jute bags for only 40 ± 2.0 h yielded low maximal fermentation temperatures Tmax (31.1 ± 0.4 °C and 37.6 ± 1.8 °C), leading to bitter, astringent, woody, and earthy cocoa liquor. Longer fermentation (63 ± 6 h) in wooden boxes with turning (Wt) and in jute bags with pre-drying and turning (Jpt) achieved the highest Tmax of 46.5 ± 2.0 °C, and a more acidic cocoa liquor, particularly in Wt (both locations) and Jpt (location E). Therefore, it is recommended to ferment for a minimum duration from day 1 to 4 (63 ± 6 h), whether using plastic bags (with mandatory pre-drying) or jute bags (with or without pre-drying or turning). Furthermore, this study underscores the risks associated with excessively high drying temperatures (up to 95.2 ± 13.7 °C) and specific dryer types, which can falsify cut-tests and introduce unwanted burnt-roasted off-flavors in the cocoa liquor.

## 1. Introduction

Cocoa beans, the principal raw material for chocolate, first undergo a fermentation and drying step in the country of origin before further processing to, e.g., chocolate [1,2].

Therefore, the cocoa pods are opened in the field, and the fermentation of the pulp-bean mass starts with a succession of activities of naturally present yeasts, lactic acid bacteria (LAB), and acetic acid bacteria (AAB) [3,4].

Numerous microbiological and physicochemical processes occur during the fermentation process. These processes primarily aim to reduce bitterness and astringency while generating essential aroma precursors that subsequently react during the later roasting process, contributing to the desired characteristic cocoa flavor [3,5,6].

It is already widely known that many parameters influence the post-harvest process and, therefore, the cocoa bean and chocolate quality. In the past, various studies were carried out to investigate these influences specifically, such as the ripeness of the cocoa pods, pod storage, pre-conditioning (pre-drying and depulping), environmental contamination, batch sizes during fermentation, fermentation time, turning time and frequency during fermentation, fermentation device, and drying method (natural or artificial drying, dryer type, turning frequency, drying temperature) [7,8,9,10,11,12,13,14,15,16,17,18,19,20,21,22,23,24,25,26].

It is also known that factors such as the cocoa variety, soil type, age of cocoa trees, prevailing weather conditions, growth location, seasonal variations, the separation of healthy and infected pods prior to pod opening, and whether the placenta is removed before fermentation, all exert significant influences on the fermentation and drying processes and, consequently, the overall cocoa bean quality [5,27,28,29,30].

In Ecuador, the majority of cocoa producers, about 84%, operate small-sized farms, while the remaining 16% manage medium and large-sized farms. The cocoa moves from the producers (with or without the post-harvesting process) typically through middlemen (with or without the post-harvesting process) to exporters [31]. Farming practices and post-harvest processes, which are carried out mainly on small-sized farms, are often conducted using knowledge passed down through generation-to-generation experience [32,33]. This natural, mostly poorly controlled and heterogenous post-harvest process affects, in addition to the previously listed parameters, the quality and flavor of the fermented and dried cocoa beans and the chocolates produced thereof [34,35]. Therefore, fermentation and drying are crucial steps for quality, e.g., to have well-fermented beans without off-flavors [7]. Also, in order to neutralize the inconsistent quality of chocolate, chocolate manufacturers blend different cocoa batches to reach a uniform and consistent quality [35,36].

In Ecuador, various processes are applied during cocoa post-harvesting, both at the farmer and middleman levels. These processes involve the use of different fermentation devices, including wooden boxes, jute or plastic bags, plastic tubs, or heap fermentations. Additionally, some steps, such as pre-drying and turning during fermentation, are occasionally incorporated. Moreover, the fermentation duration can vary, ranging from 2 to 4 days [7,11,17]. The drying of cocoa beans is carried out either artificially using various dryer types, some with ventilation and some without, or naturally (sun drying) on surfaces like concrete floors, roadsides, wooden or cane mats, extended plastic sheets on the ground, or drying nets, typically under a plastic canopy (observed during the current study).

Cocoa bean post-harvesting processes, including the use of different fermentation devices, and the impact of pre-drying during fermentation, fermentation durations, different geographical zones, and varying drying techniques, have already been subjects of numerous studies in Ecuador [11,37,38,39,40,41]. Nevertheless, to the best of our knowledge, this is the first comprehensive study that was carried out with cacao nacional in Ecuador to systematically investigate the combination of different parameters during fermentation (device, turning, pre-drying, and fermentation time) and drying (dryer and temperature) at two locations, influencing the cocoa bean quality. Therefore, our study aimed to gain insights into the cocoa fermentation process by conducting a total of nine different fermentation variations while considering two locations in Ecuador. These variations were defined based on common parameter combinations observed in the previous monitoring study by Streule et al. (2022) [7]. Our research included the monitoring of the fermentation and drying process, focusing on key aspects such as pH, temperature, cell counts of yeast, lactic acid bacteria, and acetic acid bacteria. Additionally, we conducted analyses of dried beans, including sugars and organic acids, cut-tests, and the pH of the cotyledon. The sensorial assessment of the cocoa bean liquor allowed us to evaluate potential influences of fermentation variations on the fermentation progress and overall bean quality, providing valuable insights into the characteristics of the final product—cocoa liquor. This comprehensive approach aimed to detect the adequate process steps for ideal cocoa fermentation.

## 2. Materials and Methods

### 2.1. Fermentation and Drying Experiments

In total, nine different post-harvest variations (Table 1) were performed at two locations (A: province Manabí; E: Los Ríos) in Ecuador between 04/2018 and 08/2018. In the following chapters, the abbreviations of the fermentation variations will be used according to Table 1: Wt (wooden box with turning), J (jute bag without pre-drying and without turning), Jp (jute bag with pre-drying), JpT (jute bag with pre-drying and higher drying temperature), JpsT (jute bag with pre-drying, shorter fermentation time, and higher drying temperature), Jpt (jute bag with pre-drying and turning), P (plastic bag without pre-drying and without turning), Pp (plastic bag with pre-drying), and PpT (plastic bag with pre-drying and higher drying temperature) at locations A (province Manabí) and E (Los Ríos).

**Figure 1:**
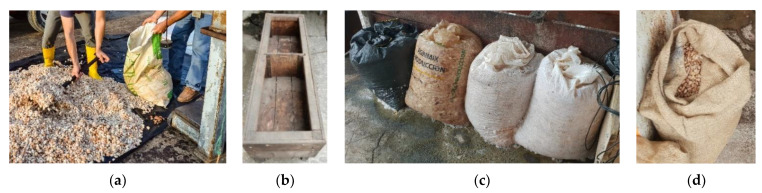
(**a**) Mixing of fresh cocoa beans from different farmers on day 1 at location A; (**b**) wooden box with slits between the boards for pulp drainage, used for variation Wt; (**c**) different used plastic bags for day 1 until day 2 for all variations except for Wt and the following days for variations P, Pp, and PpT; (**d**) used jute bags for the fermentation variations J, Jt, Jp, Jpt, JpT, and JpsT.

Cocoa Nacional pods (selected as described in [7]) were harvested. Beans were extracted on the same day or the next day at various farming locations, marking the start of the fermentation process (referred to as day 1 of fermentation). The healthy beans were then transported to the fermentation site, homogenized (e.g., Figure 1a), and subsequently divided into three to five equal approximately 50-kg samples, depending on the specific variations conducted simultaneously.

For each fermentation run, a control fermentation (Wt) was carried out using approximately 50 kg of fresh beans placed in a laurel wooden box that was divided into two compartments (one compartment measuring 0.8 m in length, 0.49 m in width, and 0.55 m in height, Figure 1b). In contrast, for all other variations, the same quantity of beans was filled separately into perforated plastic bags (approx. 50 kg), a common fermentation device outlined in Streule et al. (2022) [7]. These plastic bags were initially used for the first night of fermentation in all variations except Wt. Prior to use, they had been washed and dried, having previously transported items such as wheat flour or animal food (Figure 1c). Subsequently, the same plastic bags were used for the following fermentation days in the cases of P, Pp, and PpT.

In variation Wt, the daily turning of the beans was accomplished by relocating them to the other compartment of the box. In contrast, for the bags in variation Jpt, turning was carried out by transferring the cocoa into another bag and then placing it back into the same bag or by manually moving the beans within the same bag.

**Figure 2:**
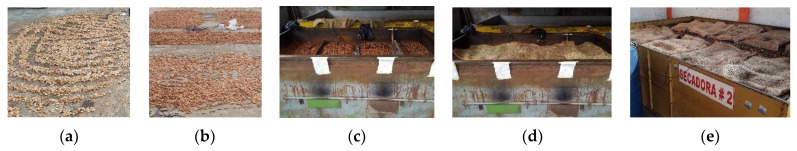
(**a**) Pre-drying on concrete floor on day 2; (**b**) sun-drying on concrete floor on day 3 or 4; (**c**) artificial drying at location A, variations separated with wooden sticks; (**d**) beans covered with jute bags during artificial drying at location A; (**e**) beans drying in jute bags on top of other cocoa beans at location E.

For variations involving pre-drying, the beans were left on a concrete floor for approximately 4 to 7 h (Figure 2a). Turning of the beans during pre-drying was based on solar radiation, with an increased frequency on sunnier days and reduced turning frequency during periods of limited sunlight. Subsequently, the cocoa was placed in either jute bags (Figure 1d) or plastic bags, depending on the specific variation (Table 1). The used jute bags were new for the first repetition and were subsequently washed and dried at the same location after each completed process.

Drying in variation JpsT commenced on day 3, with a process that involved sun-drying the beans on a concrete floor for a period ranging from 4 to 9 h (Figure 2b). Following this initial step, the beans were spread in a turned-off dryer overnight before completing the drying process on the following day in an artificial dryer located at site A.

In contrast, all other variations underwent sun-drying on day 4 for a duration of 1 to 7 h, during which the beans were spread on the floor with periodic turning. Subsequently, artificial drying with turning steps was performed according to the available dryers and their setting options.

At location A, the beans were dried directly in a dryer (Figure 2c), with jute bags used as covering (Figure 2d). To achieve different drying temperatures (“high” and “low” temperatures at location A), the gas was ignited at a very low level to maintain a low-temperature drying process. On the other hand, at location E, beans were dried inside jute bags atop other cocoa beans, with adjustments made to maintain the temperature as per common practice (Figure 2e).

Following this, the still-moist beans were left overnight in a turned-off dryer, covered with jute bags. On day 5, the objective was to reduce the humidity to less than 8% using artificial drying. If the beans had not reached this desired moisture level, they were further sun-dried on a concrete floor to complete this stage.

### 2.2. Sampling

Samples were collected daily during the fermentation process at various places in the fermentation device (according to the temperature probes’ positions, Section 2.3.1) using disinfected gloves. These mixed samples were then transported, without refrigeration, for a maximum of 30 min before initiating the analysis of microbial counts for both fresh and fermenting beans, as well as the pH of fresh, fermenting, and dried beans. Additionally, cut-tests on the beans were performed once the moisture level reached <8%. The dried beans were subsequently stored and shipped to Switzerland for the determination of sugars and organic acids and for sensory evaluation.

### 2.3. Analyses

#### 2.3.1. Measurement of Temperature

The temperature of the beans during fermentation and drying was recorded according to [7] while positioning the probes as in setup 2 during fermentation.

#### 2.3.2. Enumeration of Yeasts, Lactic Acid Bacteria, and Acetic Acid Bacteria

Cocoa bean samples during fermentation (daily) were mixed with diluent (1 g/L bacteriological peptone (HiMedia Laboratories LLC, Kennett Square, PA, USA); 8.5 g/L sodium chloride (Sigma-Aldrich, Inc., St. Louis, MO, USA)) in a ratio of 1:10 and manually kneaded for 2 min. Serial dilutions were plated using the drop-plate method [42] on yeast peptone mannitol medium (YPM), supplemented with cycloheximide and penicillin, all supplied by Sigma-Aldrich, Inc., St. Louis, MO, USA [42] for acetic acid bacteria (AAB), surface plated on 3M™ Petrifilm^®^ Lactic Acid Bacteria Count Plates for lactic acid bacteria (LAB), and for yeasts on 3M™ Petrifilm^®^ Rapid Yeast and Mold Count Plates (both from 3M Food Safety, St. Paul, MN, USA). Yeast and AAB were incubated at 26 °C for 2 to 4 days, and LAB at 37 °C for 2 to 3 days. Calculation formula for the CFU (colony forming units) per gram according to ISO 7218:2007 [43] was used after counting the CFU on the plates.

#### 2.3.3. Measurement of pH of Cotyledon and Pulp

The pH values of cotyledon and pulp were determined using indirect methods according to Romanens et al. (2018, 2020) [42,44].

#### 2.3.4. Determination of Sugars and Organic Acids in Dried Cocoa Beans

Saccharose, glucose, fructose, citric acid, lactic acid, and acetic acid were determined in the samples of runs 3 and 4 (Wt, P, Jp, Jpt, and Pp) at location E and of runs 1 and 2 (Wt, J, Jpt, JpT, JpsT, PpT, and Jp only of run 1) at location A. The samples were prepared according to Romanens et al. (2018, 2020) [42,44] with slight modifications. In this study, only the cotyledons without shells were used, and the centrifugation parameters varied: 15 min at 17,000× *g* at 4 °C. High-performance liquid chromatography (HPLC) was performed on an HPLC system (Agilent 1260). The same columns were used as described by Müller et al. (2022) [45]. Organic acids (citric acid, lactic acid, and acetic acid), saccharose, and glucose were analyzed with ROA-Organic Acid H+ column and fructose with Rezex RPM monosaccharides. A Refractive Index (RI) detector (1260 RID G1362A, Agilent, Santa Clara, CA, USA, temperature set to 50 °C) was utilized for sugar analysis and a Diode Array Detector (DAD) (1260 RID G4212B, Agilent, Santa Clara, CA, USA, wavelength set to 210 nm) for organic acid analysis.

#### 2.3.5. Cut-Test of Dried Beans

The cut-test of each sample was performed following the procedures described in Streule et al. (2022) [7].

#### 2.3.6. Sensory Assessment

For the sensory description, mixed samples from run 3 + 4 (Wt, P, Jp, Jpt, and Pp) at location E and from run 1 + 2 (Wt, J, Jp, Jpt, JpT, and JpsT) from location A were prepared. Cocoa liquor samples were prepared in accordance with the protocol detailed in Streule et al. (2022) [7]. The Lindt & Sprüngli cocoa panel (Kilchberg, Switzerland) evaluated the samples regarding flavor (basic tastes, retronasally perceived aromas, and astringency) with a generic descriptive analysis based on QDA [46]. Participation in the panel was voluntary, and assessors were selected according to ISO Norm (ISO 8586:2012) [47] and trained for the assessment of cocoa liquor. Nine assessors participated in the study. The assessors were informed of the research scope at the beginning of the study and debriefed on the results at the end. All samples underwent Lindt’s standard quality control procedure to ensure adherence to food safety standards. The anonymized setup ensured the observation of the individual’s privacy rights.

The sensory attribute list from a preceding study of post-harvest cocoa beans from Ecuador [7] was adjusted to the sample space of the current study through discussions with the panel using individual sorted napping tasks coupled with word association (described, e.g., in Lê et al. (2015) [48]) as starting point. The final attribute list is described in Table 2.

For the data collection, the panelists rated the samples in triplicate (in three separate sessions) on a 10-cm VAS scale (scale anchors in Table 2).

### 2.4. Statistics

All fermentation variations were performed between 2 and 13 times (Table 1). For normally distributed data (checked using Shapiro–Wilk test and/or Q–Q plot), a one-way ANOVA, followed by posthoc (Tukey-HSD Test), was applied per location. Sensory descriptors were tested for significant differences between samples using a Mixed ANOVA with assessor as random factor (R package “lmerTest”). For significant differences between samples, significance levels were calculated based on Tukey-HSD (R package “multcomp”), and a multivariate data analysis (PCA) was applied for sensory results.

Multivariate analysis using partial least squares (PLS) was applied to visualize relations between fermentation and drying parameters, end quality (saccharose, organic acids), and variation and location. Predictor and response variables were selected accordingly. Mean values of the runs were used. 

Statistical analyses were performed using RStudio (version 4.3.0) and the tests were performed at a significance level of *p* < 0.05.

## 3. Results

### 3.1. Microbial Counts during Fermentation

**Figure 3:**
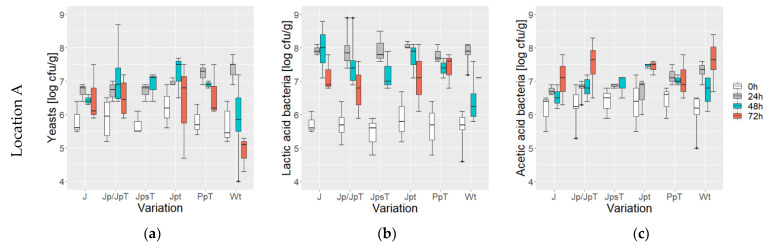

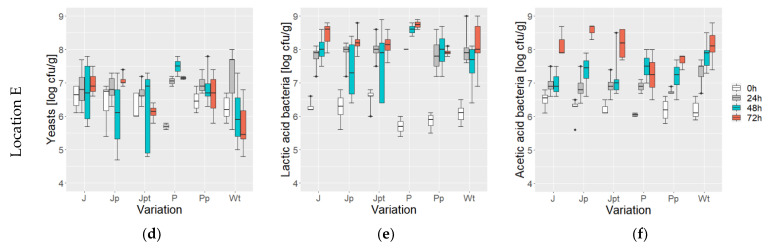
Cell counts (log cfu/g) of yeast (**a**,**d**), lactic acid bacteria (**b**,**e**), and acetic acid bacteria (**c**,**f**) at the two locations A (**a**–**c**) and E (**d**–**f**) from variations J (jute bag without pre-drying, without turning), Jp (jute bag with pre-drying)/JpT (jute bag with pre-drying and high drying temperature), JpsT (jute bag with pre-drying, shorter fermentation time and higher drying temperature), Jpt (jute bag with pre-drying and turning), P (plastic bag without pre-drying nor turning), and Pp (plastic bag with pre-drying)/PpT (plastic bag with pre-drying and high drying temperature) recorded after 0 h, 24 h, 48 h, and 72 h of fermentation; *n* = 1–7.

Microbial developments during fermentation are shown in Figure 3. Initial counts of yeasts and LAB (lactic acid bacteria) were similar within location but differed significantly between locations. Specifically, at location E, yeast counts were 6.3 ± 0.4 log cfu/g, while at location A, they measured 5.8 ± 0.5 log cfu/g (*p* < 4.423 × 10^−7^). On the other hand, AAB (acetic acid bacteria) counts were consistent at both locations, with values in the range of 6.2 ± 0.4 log cfu/g (*p* = 0.9533). Across all variations at both locations, an initial increase in yeast, LAB, and AAB counts was observed within the first 24 h. Subsequently, a notable decrease in yeast counts occurred in variation Wt at both locations, reaching values of 4.9 ± 0.5 log cfu/g at location A and 5.7 ± 0.7 log cfu/g at location E by the end of the fermentation process. Similar decreasing trends in yeast counts until the end of fermentation were also noted in variations J and PpT at location A. In contrast, in other variations, yeast counts continued to rise until 48 h before declining towards the end of fermentation. This trend was particularly prominent in variation P at location E, where yeast counts reached 7.5 ± 0.3 log cfu/g at 48 h.

Lactic acid bacteria showed a decreasing trend between 24 h and the end of fermentation in all variations at location A to a mean cell count of 7.1 ± 0.6 log cfu/g. At location A, there was a consistent decrease in lactic acid bacteria (LAB) counts between 24 h and the end of fermentation across all variations, resulting in a mean cell count of 7.1 ± 0.6 log cfu/g. Conversely, at location E, LAB counts either increased or remained stable until the completion of fermentation. These final LAB counts reached 8.2 ± 0.5 log cfu/g at location E, significantly higher than those at location A (*p* < 1.488 × 10^−5^). Notably, variation P exhibited a remarkable increase in LAB counts from 8 ± 0 log cfu/g at 24 h to 8.6 ± 0.2 log cfu/g at 48 h in the fermentation process.

At location A, acetic acid bacteria increased in all variations toward the end of fermentation. Particularly noteworthy were the similar cell counts observed in the last 24 h and 48 h in variations JpsT, Jpt, and PpT, respectively. Additionally, the progression in Wt was notable, featuring a decrease at 48 h followed by a subsequent increase until the end of the fermentation process. The average cell counts at the end of fermentation were 7.4 ± 0.6 log cfu/g for variations fermented for 72 h and 6.9 ± 0.3 log cfu/g for variation JpsT fermented for 48 h at this location.

At location E, across all variations, AAB counts increased steadily until 72 h, reaching counts of 8.2 ± 0.5 log cfu/g, with the exception of variation P, where the values remained similar in the last 24 h (ranging from 6.5 to 8 log cfu/g).

### 3.2. Temperature Development during Fermentation and Drying Temperatures

**Figure 4:**
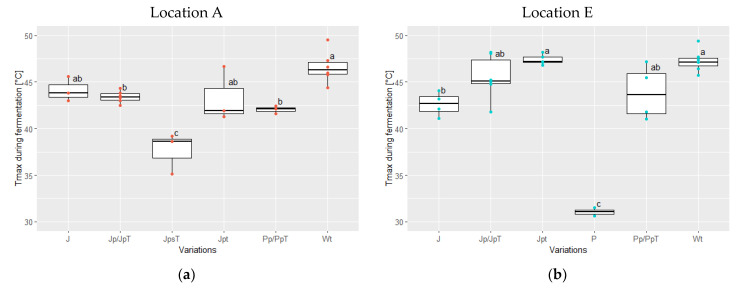
Reached maximum temperature Tmax (°C) during the fermentations at location A in red (**a**) and at location E in blue (**b**) for the following variations: J (jute bag without pre-drying, without turning; location A: *n* = 3, E: *n* = 4), Jp (jute bag with pre-drying; E: *n* = 6)/JpT (jute bag with pre-drying and high drying temperature; A: *n* = 3), JpsT (jute bag with pre-drying, shorter fermentation time and higher drying temperature; A: *n* = 3), Jpt (jute bag with pre-drying and turning; A: *n* = 3, E: *n* = 5), P (plastic bag without pre-drying nor turning; E: *n* = 2), Pp (plastic bag with pre-drying; E: *n* = 4)/PpT (plastic bag with pre-drying and high drying temperature; A: *n* = 3), and Wt (wooden box with turning; A: *n* = 6; E: *n* = 7) (ANOVA with Tukey HSD). Values that do not share the same letter differ significantly (*p* < 0.05).

During the fermentation of variations J, Jp, JpT, and Pp, PpT of both locations, the temperature increased until reaching a similar peak temperature of 43.8 ± 2.0 °C (ranging from 41.0 to 48.2 °C) (Figure 4a,b). In variations with turning (Wt) and additional pre-drying (Jpt), considering both locations, the temperature rose slightly higher, averaging 47.0 ± 1.4 °C and 45.9 ± 2.5 °C, respectively. In contrast, fermenting in plastic bags without pre-drying and turning at location E (P) did not result in a temperature increase, yielding the significantly lowest Tmax of 31.1 ± 0.4 °C (*p* < 0.05). But while adding a pre-drying step, a Tmax of 43.9 ± 3.0 °C was reached for Pp (location E). Additionally, fermenting for one day less, i.e., 3 days (40 ± 2 h) instead of 4 days (63 ± 6 h), with jute bags and the pre-drying step (JpsT) at location E resulted in a significantly lower temperature increase, reaching 37.6 ± 1.8 °C, compared to all other fermentations except P (*p* < 0.05).

When comparing the variations involving jute and plastic bags with the same fermentation duration, regardless of the location, it was found that the Tmax was reached significantly faster (*p* = 0.002) when pre-drying was performed (48 ± 8 h; variations Jp/JpT, Jpt, Pp/PpT) compared to when it was not implemented (56 ± 7 h; variations J, P) (Figure S1).

At location A, the beans were dried in a dryer with direct contact with fire and no ventilation, which resulted in reaching maximum temperatures of up to 95.2 ± 13.7 °C (Table 3). In contrast, at location E, the beans were dried inside jute bags placed atop other cocoa beans, utilizing a dryer with indirect contact to fire equipped with ventilation. This approach led to significantly lower maximum (74.7 ± 6.5 °C) and average temperatures (48.0 ± 4.2 °C) compared to those at location A (*p* < 0.05). 

At location A, where the drying temperature was varied in some samples, an average temperature during drying of 64.3 ± 14.7 °C was reached. Samples Wt, J, Jp, and Jpt were dried at an average temperature of 59.0 ± 12.8 °C. In contrast, PpT, JpT, and JpsT were dried at a significantly higher average temperature of 73.2 ± 13.8 °C (*p* = 0.02384). The larger standard deviation of 13.8 °C in the second group can be attributed to the lower drying temperatures measured in the case of sample JpsT (Table 3). No significant differences were observed in the maximum drying temperatures across these two sample groups (*p* > 0.05).

### 3.3. Pulp and Cotyledon pH

**Figure 5:**
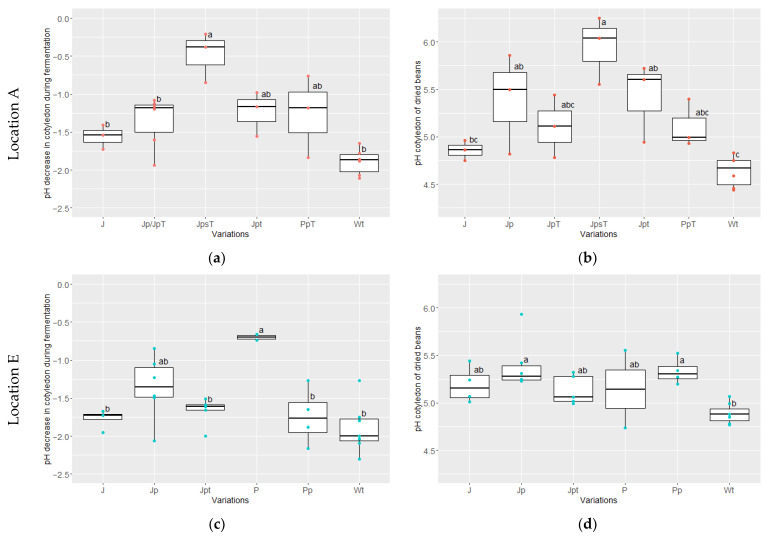
pH decrease in cotyledon during fermentation (pH final–pH initial) at location A (**a**) and E (**c**); pH cotyledon of dried beans at location A in red (**b**) and E in blue (**d**) for the following variations: J (jute bag without pre-drying, without turning; location A: *n* = 3, E: *n* = 4), Jp (jute bag with pre-drying; E: *n* = 6)/JpT (jute bag with pre-drying and high drying temperature; A: *n* = 3), JpsT (jute bag with pre-drying, shorter fermentation time and higher drying temperature; A: *n* = 3), Jpt (jute bag with pre-drying and turning; A: *n* = 3, E: *n* = 5), P (plastic bag without pre-drying nor turning; E: *n* = 2), Pp (plastic bag with pre-drying; E: *n* = 4)/PpT (plastic bag with pre-drying and high drying temperature; A: *n* = 3), and Wt (wooden box with turning; A: *n* = 6; E: *n* = 7) at locations A (red) and E (blue) (ANOVA with Tukey HSD). Values that do not share the same letter differ significantly (*p* < 0.05).

At both locations, the initial pH values in the cotyledons were within the range of 6.3 to 6.8. However, the evolution of cotyledon pH during fermentation differed among some variations (Figure 5). Notably, variations JpsT (at location A, Figure 5a) and P (at location E, Figure 5c) exhibited a rather lower pH development (delta of −0.9 to −0.2), with end-of-fermentation pH values of 6.0 ± 0.2 and 5.90 ± 0.03, respectively. In contrast, the pH in the Wt fermentation dropped to 4.6 ± 0.2 (considering both locations), resulting in a higher delta of −1.8 to −2.3 compared to variations JpsT, P, and Jp/JpT. Nevertheless, the pH values for variations J, Jp, JpT, Jpt, Pp, PpT, and Wt (of both locations) all decreased within the same pH range of 0.8–2.9, ultimately reaching a pH of 4.9 ± 0.3.

After the drying process, pH values in the cotyledon increased in 75% of the measurements compared to the end-fermentation values (data not shown). Remarkably, variation JpsT at location A (short fermentation in jute bags with pre-drying) exhibited a rather higher pH range of 5.6–6.3 (Figure 5b), in contrast to the other variations (including variations at location E), which averaged at 5.1 ± 0.3.

**Figure 6:**
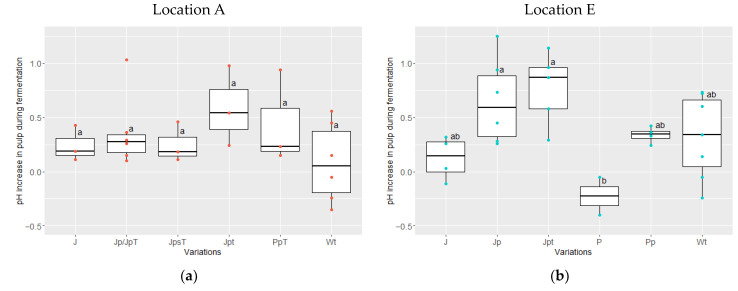
pH increase in pulp during fermentation (pH final–pH initial) at location A in red (**a**) and E in blue (**b**) for the following variations: J (jute bag without pre-drying, without turning; location A: *n* = 3, E: *n* = 4), Jp (jute bag with pre-drying; E: *n* = 6)/JpT (jute bag with pre-drying and high drying temperature; A: *n* = 3), JpsT (jute bag with pre-drying, shorter fermentation time and higher drying temperature; A: *n* = 3), Jpt (jute bag with pre-drying and turning; A: *n* = 3, E: *n* = 5), P (plastic bag without pre-drying nor turning; E: *n* = 2), Pp (plastic bag with pre-drying; E: *n* = 4)/PpT (plastic bag with pre-drying and high drying temperature; A: *n* = 3), and Wt (wooden box with turning; A: *n* = 6; E: *n* = 7) (ANOVA with Tukey HSD). Values that do not share the same letter differ significantly (*p* < 0.05).

On the other hand, the measured initial pH of the pulp was significantly lower (*p* = 0.000127) at location A (ranging from 3.6 to 4.0) than at E (3.8 to 4.7). On average, the difference between the final and initial pH of the pulp during fermentation increased in all variations, except in the case of P at location E (Figure 6b). In variation P, the pH of the pulp decreased from a range of 4.02–4.1 to a range of 3.97–3.7. During certain fermentation runs, there were instances where the pH of variation Wt (wooden boxes with turning; in three runs at location A and two runs at location E) and variation J (jute without pre-drying, without turning; in one run at location E) also showed an increase.

### 3.4. Sugars and Organic Acids in Dried Cocoa Beans

At location E, samples from variations Jp, Jpt, and Pp exhibited similar saccharose contents, ranging from 4.1 to 10.9 mg/g, along with comparable glucose and fructose concentrations (Table 4). However, in variation Wt, sugars were only detected in one out of three measured samples. Variation P, on the other hand, exhibited a higher saccharose content, a lower glucose level, and a fructose concentration similar to Jp, Jpt, and Pp. At location A, the saccharose contents generally exceeded those observed at location E. Notably, samples PpT and JpsT exhibited even higher saccharose levels, reaching up to 17.5 ± 3.5 mg/g and 18.4 ± 1.2 mg/g, respectively. In contrast, cocoa fermented in a wooden box (variation Wt) showed lower saccharose content at 6.1 ± 2.9 mg/g. The glucose values across all samples fell within a similar range of 0.0–3.4 mg/g, while fructose levels displayed a broader but still comparable spectrum, ranging from 1.2 to 10.1 mg/g.

At location E, all citric acid values fell within a consistent range, showing no notable variations. In contrast, variation P demonstrated higher lactic acid values at 8.6 ± 1.6 mg/g, than the other samples ranging from 1.2 to 4.9 mg/g. The highest acetic acid contents, reaching 49.3 mg/g and 46.1 mg/g, were detected in single samples of variations Wt and Pp, respectively, while the remaining results were similar.

Overall, at location A, organic acid contents were lower than those measured at location E. Citric acid and lactic acid levels remained similar across all variations. However, in the case of sample JpsT, the citric acid content slightly exceeded the others, measuring 4.6 and 5.8 mg/g. Additionally, acetic acid content showed uniformity across all variations, with the lowest value recorded at 3.3 mg/g in sample Jp.

### 3.5. Cut-Test of Dried Beans

No fermentation variation showed significant differences in categories such as well-fermented, slightly fermented, violet, slaty, or moldy beans (*p* > 0.05). However, there were notably more well-fermented beans at location A than at location E (*p* = 0.00141), as seen in Table 5. Conversely, location E had a higher count of slightly fermented (*p* = 0.005626) and violet beans (*p* = 0.01492) than location A, regardless of the fermentation method used.

### 3.6. Sensory Description of Cocoa Bean Mass

All sensory descriptors, with the exception of the attribute “nutty”, discriminated samples significantly (Table 6). For some descriptors (“fruity, banana”, “floral”, and “spicy”), all samples were placed on the same significance level with the Tukey HSD posthoc test, indicating only a weak discrimination between samples. 

**Figure 7:**
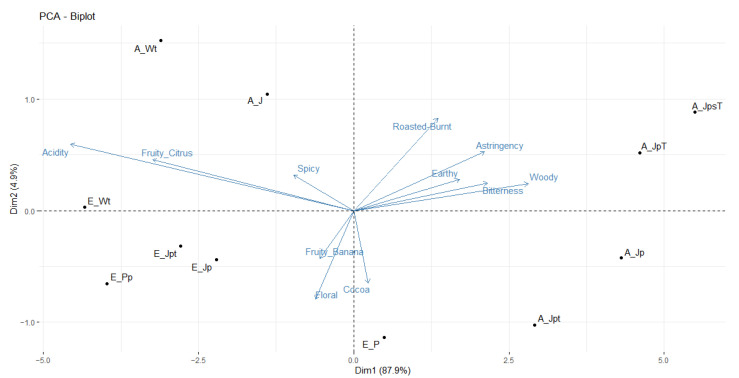
Principle component analysis (PCA) based on sensory descriptors that discriminated samples (J (jute bag without pre-drying, without turning), Jp (jute bag with pre-drying), JpT (jute bag with pre-drying and high drying temperature), JpsT (jute bag with pre-drying, shorter fermentation time and higher drying temperature), Jpt (jute bag with pre-drying and turning), P (plastic bag without pre-drying nor turning), Pp (plastic bag with pre-drying), and Wt (wooden box with turning) at locations A and E) significantly.

A PCA based on the unstandardized sensory descriptors that discriminated the samples significantly is displayed in Figure 7. The first dimension explained 87.9% of the variance with “acidity” and “fruity (citrus)” pointing in one direction (left side) and “bitterness”, “astringency”, “woody”, and “earthy” in the opposite (right side). The reference fermentations Wt from both locations (A_Wt and E_Wt) that were fermented in boxes were placed on the left-hand side of the plot. Sample location on dimension 1 further reveals differences between locations, with location E generally being more oriented towards sensory descriptors on the left-hand side (e.g., acidity) and location A being more oriented towards descriptors on the right-hand side (e.g., bitterness). The second dimension explained only 4.9% of the variance separating samples according to other flavor attributes. Samples JpT and JpsT at location A were rated high in “roasted-burnt”. On the opposite side were the flavor attributes “cocoa”, “floral”, and “fruity, banana”. Sample Jpt at location A was rated significantly higher regarding “cocoa” than other samples (E_Jpt, A_JpsT), while no significance levels could be found for “floral” and “fruity, banana”.

### 3.7. Multivariate Analysis Using Partial Least Squares (PLS)

#### 3.7.1. Effect of Post-Harvesting Parameters on Selected Quality Parameters

**Figure 8:**
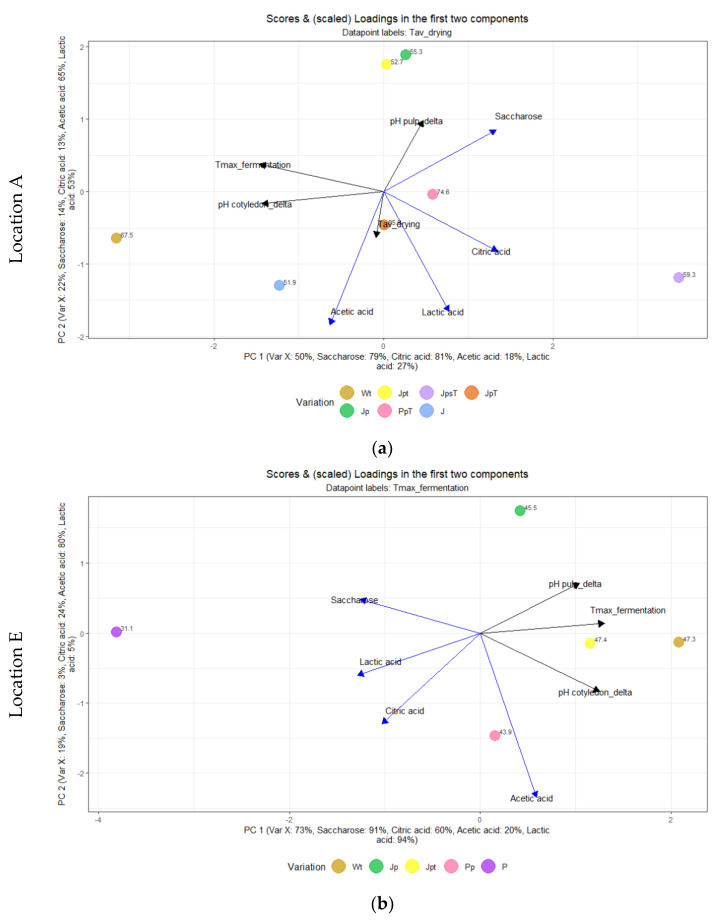
(**a**) Partial least squares (PLS) of predictors (black; Tmax during fermentation, Tav during drying, pH cotyledon_delta between initial and end of fermentation, and pH pulp_delta between initial and end of fermentation) and response variables (blue; Citric acid, Lactic acid, Acetic acid, and Saccharose) of fermentation variations J, Jp, JpsT, Jpt, JpT, PpT, and Wt at location A; (**b**) Partial least squares (PLS) of predictors (black; Tmax during fermentation, pH cotyledon_delta between initial and end of fermentation, and pH pulp_delta between initial and end of fermentation) and response variables (blue; Citric acid, Lactic acid, Acetic acid, and Saccharose) of fermentation variations Jp, Jpt, P, Pp, and Wt at location E.

A PLS was constructed to illustrate the influences of the fermentation variations on fermentation and, therefore, on the end bean quality (saccharose, organic acids). At location A (Figure 8a), the data points were labeled with the average drying temperature due to sample groups (samples PpT, JpsT, and JpT) intended to dry at higher temperatures. The first PC explained 50%, and the second PC explained 22% of the variance for location A (Figure 8a). It is worth noting that variations Wt and JpsT are positioned in opposite directions. The predictor variable Tmax during fermentation is especially strongly associated with Wt, while saccharose, citric acid, and lactic acid as responsive variables are more closely related to the sample JpsT. Variations Jp and Jpt are close together. At location E (Figure 8b), the first PC accounted for 73% of the variance, and the second PC explained 19%. A similar pattern emerges with opposing variations Wt and P at location E.

#### 3.7.2. Effect of Drying Temperature on Cut-Test and Acetic Acid per Location

**Figure 9:**
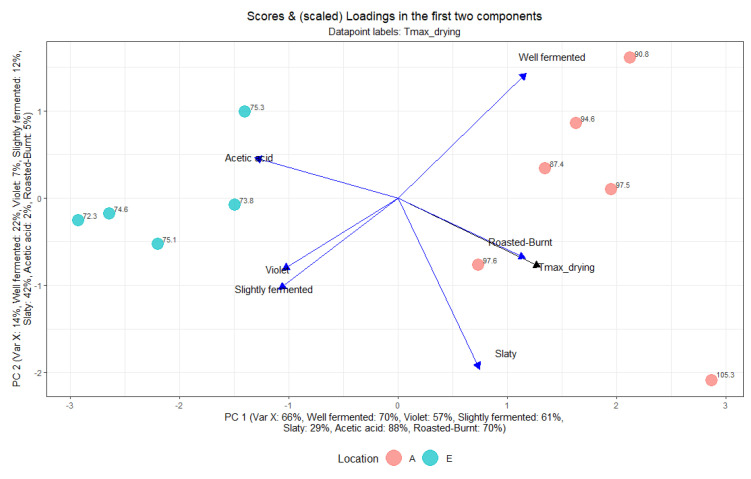
Partial least squares (PLS) of predictor (black; Tmax during drying) and response variables (blue; Well fermented, Slaty, Violet, Slightly fermented, Acetic acid, and Roasted-Burnt) at locations A and E.

The effect of the drying temperature on the cut-test and acetic acid content of dried beans is illustrated in the PLS in Figure 9. Especially maximal temperatures during drying resulted in well-fermented beans (70%) and roasted-burnt cocoa liquor (70%), which applies to the averages of all the variations of the same location A. On the other hand, lower Tmax during drying resulted in less slightly fermented beans, which were the variations at location E. Also, the variable acetic acid content of dried beans points in the opposite direction, towards location E, where the beans were dried at lower temperatures.

## 4. Discussion

### 4.1. Different Microbiological and Physicochemical Dynamics and Quality Depending on Fermentation Variation

In this study, we explored various fermentation variations, which differed in terms of the fermentation device, steps during fermentation (such as pre-drying and turning), fermentation duration, and drying temperature. Our investigation aimed to uncover their effects on microbial dynamics, temperature profiles, pH values, and the overall quality of the end product, which includes factors like sugars, organic acids, sensory, and cotyledon pH. We observed significant differences in these factors.

Regarding microbiological dynamics, we observed a remarkable increase in yeast population at 48 h, particularly in variation P at location E. Additionally, variation P exhibited the lowest maximal fermentation temperature (*p* < 0.05), which was accompanied by limited growth of acetic acid bacteria (AAB) after 72 h. Yeasts are known to have poor tolerance to increasing temperatures [49,50,51,52]. This explains the relatively high yeast cell counts observed until the end of fermentation in variation P. Furthermore, we found a significant negative correlation between the maximum temperature and yeast counts after both 48 h (r = −0.46, *p* < 0.05) and 72 h (r = −0.44, *p* < 0.05).

In contrast, yeasts and AAB exhibited similar patterns in fermentation JpsT (location A) as compared to Jp/JpT (considering both locations) until 48 h, after which a decline in yeast population and an increase in AAB were observed in the longer fermentations (Jp/JpT) during the last 24 h. These observations, along with the significantly lower maximum temperature during fermentation compared to Jp/JpT, indicate that a fermentation period of 3 days (40 ± 2 h) may not be sufficient. Typically, during fermentation, the cocoa bean mass reaches temperatures of 45 to 50 °C [27,36,53,54]. In our study, all variations, except for JpsT and P, reached temperatures in the range from 41.0 to 49.5 °C.

The variations JpsT (location A) and P (location E) exhibited similar trends in cotyledon pH development until the end of fermentation, mirroring the patterns observed in maximum temperature (Tmax), indicating a potentially weak fermentation. This negative correlation (r = −0.66, *p* < 0.05) between cotyledon pH at the end of fermentation and Tmax was previously documented also in Streule et al. (2022) [7]. Consequently, we observed that a higher cotyledon pH at the end of fermentation, coupled with a lower Tmax during fermentation, resulted in a weaker fermentation. This weaker fermentation implied, e.g., reduced degradation of saccharose in the cotyledon, resulting in higher scores for sensory attributes such as bitterness or astringency, typically associated with weak fermentation. Notably, when pre-drying was implemented for the plastic bag fermentation, or when the fermentation duration was extended by one day, we observed a higher Tmax and lower cotyledon pH. Regarding jute bag fermentations with and without pre-drying, no significance can be seen, only tendencies.

In contrast, variation Wt (and Jpt at location E) exhibited the highest recorded Tmax compared to the other fermentation variations, accompanied by a drop in cotyledon pH to relatively low values (especially variation Wt). The resulting cocoa liquor was highly rated for attributes such as “acidity” and “fruity, citrus”, indicating a high degree of fermentation. This suggests that organic acids (e.g., acetic acid) were produced during fermentation and diffused into the cotyledon, lowering the pH and triggering enzymes. This enzymatic activity, possibly due to subcellular seed structure destruction, contributes to the development of color and flavor in fermented beans [3,4,36]. Notably, these acids partially remained in the beans even during drying.

Remarkably, variations JpsT (location A) and P (location E), characterized by low Tmax and minimal changes in cotyledon pH during fermentation, exhibited the highest scores (within the location) in attributes associated with a low fermentation degree, such as bitterness, astringency, woody, and earthy flavors. Reducing bitterness and astringency is one of the purposes of cocoa fermentation [36]. Additionally, sensory evaluations showed improvements when fermentation lasted from day 1 to day 4 (63 ± 6 h), particularly when combined with pre-drying in plastic bags.

Furthermore, an interesting observation was made regarding the saccharose content. Variation JpsT exhibited the highest average saccharose content within location A, while sample P had the highest average at location E. This disparity in saccharose content further indicated a weak fermentation [55]. Considering the mentioned parameters, a low fermentation degree is linked to a weak or too short fermentation in this study. It is important to note that the cut-test results, commonly used to assess fermentation degree [53], could not be relied upon in our study due to potential falsification caused by high drying temperatures and, probably, by the type of dryer that has direct contact with fire (see Section 4.2).

Variation P, in particular, stood out with the highest score in the “floral” attribute. This could be ascribed to the higher yeast and LAB cell counts observed after 48 and 72 h of fermentation, suggesting their role in producing volatile organic compounds like esters, alcohols, and aldehydes associated with the floral attribute [56,57]. It is worth noting that the variation P also had higher lactic acid levels in the samples, which aligns with the increased growth of lactic acid bacteria. We hypothesize that these compounds, e.g., alcohols, may have survived the drying process. Interestingly, the floral attribute was more pronounced (though not significantly) at location E, possibly due to lower drying temperatures than at location A, favoring the volatilization and destruction of these compounds [58,59].

In particular, samples JpT and JpsT at location A were subjected to higher average drying temperatures, likely due to the elevated gas levels during drying and using a dryer without a ventilator. This could explain the significantly higher “roasted-burnt” flavor attribute in these samples.

### 4.2. Different Microbiological and Physicochemical Dynamics and Quality Depending on Location

Differences in the results were observed, and these variances may be attributed more to the geographical location than the specific fermentation variations. This consideration should be taken into account for future studies that aim to compare results across various locations.

In general, it was noted that organic acid levels were lower at location A compared to location E. This discrepancy could be linked to variations in microbial growth, with location A having lower initial cell counts. Furthermore, LAB decreased notably in the last 24 h of fermentation at location A, resulting in significantly lower counts than at location E (*p* = 1.488 × 10^−5^). Similarly, the growth of AAB was substantially lower after 72 h at location A than at location E (*p* = 0.0003067). These observations suggest that lower microbial activity may have led to reduced organic acid production.

Additionally, saccharose content was generally higher at location A than at location E. This implies that there was less enzymatic activity, possibly from invertase. The growth of AAB may have indirectly influenced the hydrolysis or invertase activity. These findings may explain the prevalence of woody, astringent, and bitter flavor profiles in cocoa liquor at location A, as opposed to the more acidic and fruity profiles observed at location E. The study by Tigrero-Vaca et al. (2022) [56] might confirm this explanation while finding that lab-scale fermentation with AAB was associated with fruity notes during fermentation.

Hence, the geographic location appears to have a high impact on cocoa bean quality, particularly with regard to sensory characteristics. These variations may be attributed to differing climatic conditions between locations A and E, genetic diversity in the cocoa material, and the different dynamics and interactions of microbial communities. These and further parameters contribute to the ultimate flavor profile of cocoa [27,60].

At location A, the beans were subjected to direct contact with fire during drying, which likely contributed to the higher average and maximal drying temperatures, up to 95.2 ± 13.7 °C. In contrast, at location E, cocoa beans were dried in jute bags placed on top of other cocoa beans, using a dryer with indirect contact to fire, resulting in lower average drying temperatures (48.0 ± 4.2 °C) compared to location A (64.3 ± 14.7 °C). Similar findings of higher temperatures at location A were previously reported by Streule et al. (2022) [7].

We observed that more well-fermented beans were present at location A, regardless of the fermentation method used. This observation significantly correlated with the higher drying temperatures (Tmax) (*p* < 0.05) and fewer violet beans (*p* < 0.05). These results support the assumption that high drying temperatures, potentially intensified by the use of a dryer with direct fire contact, may lead to falsifications in the cut-test results, as previously suggested in Streule et al. (2022) [7].

Furthermore, as already mentioned, the acetic acid content in dried beans was significantly lower at location A than at location E (*p* < 0.001). One plausible explanation for this difference could be the excessively high drying temperatures, resembling a roasting process (average Tmax during drying was 95.2 ± 13.7 °C at location A), which might cause the volatile acetic acid to evaporate during thermal treatment. Previous studies have documented that rapid drying and artificial drying methods led to increased acidity in beans [20,61,62,63]. But, it is worth noting that acetic acid levels tend to decrease significantly during roasting, as indicated by Frauendorfer and Schieberle’s studies [64,65] using roasting parameters of 14 min at 95 °C.

Another possible factor contributing to this difference could be the higher counts of acetic acid bacteria after 72 h and of lactic acid bacteria (assumed to be primarily heterofermentative) at location E, resulting in the production of acetic acids. These LAB are known to occur in later stages of fermentation [36,66]. Further investigations, such as measuring organic acid levels at the end of fermentation, are warranted to confirm or refute this assumption.

In conclusion, our findings indicate that drying temperatures, especially when reaching up to 100 °C without proper ventilation, can lead to a roasted-burnt flavor in cocoa beans. In contrast, temperatures up to 85 °C in a dryer with adequate ventilation did not result in cocoa beans with roasted-burnt off-flavors. However, it is worth noting that other studies have suggested an optimal drying temperature of 70 °C (e.g., Rodriguez-Campos et al. [67]).

## 5. Conclusions

Our findings emphasize the complex interplay of factors in cocoa bean post-harvesting processes and highlight the need for the careful control of fermentation parameters to achieve desired flavor profiles.

It was considered important if the fermentation is carried out in jute bags or plastic bags and that it lasts at least from day 1 to day 4 (63 ± 6 h) since the trial to stop the fermentation after day 3 in jute bags (JpsT) did not reveal sufficient temperature increase. Further, these JpsT samples were evaluated with rather high levels of bitterness, woodiness, earthiness, and astringency, which are attributes related to weak fermentation. In contrast, fermentation in wooden boxes most probably would have been completed after day 3 (40 ± 2 h), considering the temperature increase and pH decrease in cotyledon and acidic beans in sensory profiling. To confirm this assumption, further experiments with less fermentation time would be necessary.

Further, if plastic bags are used for fermentation, a pre-drying step would be mandatory to reach acceptable temperatures during fermentation and an improved sensorial profile (less bitterness and less astringency). Nevertheless, a pre-drying step needs extended knowledge because if carried out too long, the beans might dry too much, and, consequently, insufficient substrate for the microorganisms would be available. The turning was only tested in combination with a pre-drying step and would not be mandatory. Also, there were no significant differences in the jute bag fermentations with and without pre-drying, but there was a tendency to have better results with pre-drying. Nevertheless, if a turning step without pre-drying would improve the quality, it is not established and has to be tested in further trials.

Ideally, the temperature during fermentation should be controlled with a thermometer. The temperature should reach more than 40 °C to obtain an acceptable end bean quality. Nevertheless, this conclusion should be checked in further experiments during colder months in Ecuador and other zones. The monitoring of the drying temperature with a thermometer is also highly recommended; a dryer with a ventilator should be used, and the temperature should be reasonably adjusted so as not to burn the beans. 

## Figures and Tables

**Table 1:** Overview of investigated post-harvest process variations.

Fermentation Variation	Total Runs ^1^ per Location	Device ^2^	Pre-Drying on Day 1	Turning on Day 2	Drying Temperature ^3^	Fermentation Time ^4^
**Wt (control)**	A: *n* = 6, E: *n* = 7	Wooden Box	without	with ^5^	low	d1–d4
**J**	A: *n* = 3, E: *n* = 4	Plastic + Jute	without	without	low	d1–d4
**Jp**	A: *n* = 3, E: *n* = 6	Plastic + Jute	with	without	low	d1–d4
**JpT**	A: *n* = 3	Plastic + Jute	with	without	high	d1–d4
**JpsT**	A: *n* = 3	Plastic + Jute	with	without	high	d1–d3
**Jpt**	A: *n* = 3, E: *n* = 5	Plastic + Jute	with	with	low	d1–d4
**P**	E: *n* = 2	Plastic + Plastic	without	without	low	d1–d4
**Pp**	E: *n* = 4	Plastic + Plastic	with	without	low	d1–d4
**PpT**	A: *n* = 3	Plastic + Plastic	with	without	high	d1–d4

^1^ Time independent runs. ^2^ When two devices are listed, the first was used for the first night, followed by the second. If only one device is mentioned, it was used throughout. ^3^ Drying temperature at location A (high) and E (low) according to the middlemen adjustments; for lower drying temperature at location A, the gas level was tried to lower compared to the normal adjustments. ^4^ Fermentation time d1 is defined as 0 h of fermentation, d3 as 40 ± 2 h, and d4 as 63 ± 6 h. ^5^ Beans were turned also at day 3.

**Table 2:** Definition of flavor attributes and scales.

Attribute	Definition	Scale
Acidic	Basic taste, triggered, e.g., by citric, acetic, or lactic acid solution	Very weak–very strong
Bitter	Basic taste, triggered, e.g., by caffeine solution	
Fruity-Citrus	Aroma reminiscent of citrus fruits such as lemon or orange	
Fruity-Banana	Aroma reminiscent of banana (dried)	
Floral	Aroma reminiscent of dried flowers (hay flower), tea (orange blossom), or orange blossom soap	
Nutty	Aroma reminiscent of unroasted nuts with or without skins as well as raw cocoa bean (without pungent acidity)	
Woody	Aroma and mouthfeel reminiscent of wet wood (wooden ice cream sticks)	
Spicy	Aroma reminiscent of pepper	
Cocoa	Aroma reminiscent of dark chocolate	
Roasted-burnt	Aroma reminiscent of toasted/slightly burnt cacao nibs or burnt bread	Not roasted–roasted-burnt
Earthy	Aroma that is musty, reminiscent of wet earth	Very weak–very strong
Astringent	Dry, rough, or furry mouthfeel on the tongue and palate, triggered, e.g., by alum or tannin solution	

**Table 3:** Drying temperatures (maximal measured temperature Tmax and average temperature Tav) at location A and E and from lower (Wt, J, Jp, and Jpt) and higher dried (PpT, JpT, and JpsT) samples at location A.

Location/Samples	Tmax during Drying ^1^	Tav during Drying ^2^
A	95.2 ± 13.7 °C	64.3 ± 14.7 °C
E	74.7 ± 6.5 °C	48.0 ± 4.2 °C
A_Wt, J, Jp, Jpt	94.5 ± 15.3 °C	59.0 ± 12.8 °C
A_PpT, JpT, JpsT	96.3 ± 11.3 °C	73.2 ± 13.8 °C

^1,2^ *p* < 0.05.

**Table 4:** Sugars (Saccharose, Glucose, Fructose) and organic acids (citric acid, lactic acid, acetic acid) in mg/g in dried cocoa bean samples from variations J (jute bag without pre-drying, without turning; location A: *n* = 2), Jp (jute bag with pre-drying; A: *n* = 1, E: *n* = 2)/JpT (jute bag with pre-drying and high drying temperature; A: *n* = 2), JpsT (jute bag with pre-drying, shorter fermentation time and higher drying temperature; A: *n* = 2), Jpt (jute bag with pre-drying and turning; A, E: *n* = 2), P (plastic bag without pre-drying nor turning; E: *n* = 2), Pp (plastic bag with pre-drying; E: *n* = 2)/PpT (plastic bag with pre-drying and high drying temperature; A: *n* = 2), and Wt (wooden box with turning; A, E: *n* = 2) at locations A and E.

Location	Variation	Saccharose	Glucose	Fructose	Citric Acid	Lactic Acid	Acetic Acid
A	Wt	6.1 ± 2.9	2.4 ± 0.8	7.5 ± 2.6	3.3 ± 0.2	1 ± 0.1	14.8 ± 3.4
	J	10.1 ± 0.6	2.5 ± 0.8	5.5 ± 1	3.5 ± 0.0	2 ± 0.7	15.7 ± 1.6
	Jp	17.4	0.9	1.2	3.5	1.2	3.3
	Jpt	15.3 ± 1.0	2.3 ± 1.1	5.9 ± 1.9	3.6 ± 0.4	1 ± 0.1	7.6 ± 1.2
	JpT	13.8 ± 2.2	1.1 ± 1.1	3.3 ± 2.1	3.8 ± 0.6	2 ± 0.6	9.8 ± 2.9
	PpT	17.5 ± 3.5	1.6 ± 0.2	5.3 ± 0.1	4.1 ± 0.4	1.4 ± 0.2	12.9 ± 3.9
	JpsT	18.4 ± 1.2	1.8 ± 0.2	3.3 ± 1.3	5.2 ± 0.6	2.2 ± 0.2	10.1 ± 6.3
E	Wt	1.9 ± 1.9	0.7 ± 0.7	5.2 ± 5.2	13.5 ± 4.4	1.9 ± 0.7	36.6 ± 12.7
	Jp	8.6 ± 2.2	3.3 ± 0.5	8.2 ± 0.9	15 ± 7.1	2.7 ± 1.4	24.1 ± 6.2
	Jpt	6.2 ± 2.1	3.4 ± 0.4	7.8 ± 1.4	17.9 ± 2.5	2.6 ± 1.3	35 ± 0.6
	P	14.2 ± 0.8	1.7 ± 0.8	8.8 ± 1.5	21.2 ± 2.6	8.6 ± 1.6	27.9 ± 9.6
	Pp	7 ± 1.2	2.9 ± 0.0	8.1 ± 1.4	19.8 ± 0.1	4.8 ± 0.1	40.3 ± 5.8

**Table 5:** Cut-test performed with 100 dried beans from variations J (jute bag without pre-drying, without turning; location A: *n* = 3, E: *n* = 4), Jp (jute bag with pre-drying; E: *n* = 6)/JpT (jute bag with pre-drying and high drying temperature; A: *n* = 3), JpsT (jute bag with pre-drying, shorter fermentation time and higher drying temperature; A: *n* = 3), Jpt (jute bag with pre-drying and turning; A: *n* = 3, E: *n* = 5), P (plastic bag without pre-drying nor turning; E: *n* = 2), Pp (plastic bag with pre-drying; E: *n* = 4)/PpT (plastic bag with pre-drying and high drying temperature; A: *n* = 3), and Wt (wooden box with turning; A: *n* = 6; E: *n* = 7) at locations A and E. The beans were classified as well-fermented, slightly fermented, violet, slaty, or moldy. Well-fermented and slightly fermented values (%) are colored from green to red according to the highest and lowest mean, respectively. Violet, slaty, and moldy values (%) are colored from green to red according to the lowest and highest mean, respectively.

Location	Variation	Well-Fermented	Slightly Fermented	Violet	Slaty	Moldy
A	Wt	62.7 ± 27	30.5 ± 27	6.2 ± 7	0.7 ± 1.1	0
	J	62.7 ± 32.4	24.3 ± 22.9	12 ± 9.8	1 ± 1.4	0
	Jp	55.3 ± 29	27.7 ± 17.1	10 ± 13.4	3.7 ± 2.6	0
	Jpt	72.3 ± 20.2	20 ± 13.6	7.3 ± 7.1	0.3 ± 0.5	0
	JpT	56 ± 23.3	37.3 ± 16.4	6 ± 7.1	0.7 ± 0.9	0
	PpT	61 ± 28.6	33 ± 25.2	3 ± 4.2	0.3 ± 0.5	0
	JpsT	39 ± 29.7	53.3 ± 25	7.3 ± 9	0.3 ± 0.5	0
E	Wt	34.4 ± 17.7	48.4 ± 19.4	16 ± 12.1	0.3 ± 0.5	0.1 ± 0.3
	J	28 ± 9.1	51.8 ± 12.8	18.8 ± 14.5	1 ± 1.7	0.3 ± 0.4
	Jp	31.5 ± 12.6	53 ± 15.8	15.3 ± 13.1	0.2 ± 0.4	0
	Jpt	49.4 ± 12.7	39.8 ± 16.3	10 ± 5.8	0.4 ± 0.5	0.2 ± 0.4
	P	42.5 ± 24.5	41 ± 12	15.5 ± 11.5	0.5 ± 0.5	0.5 ± 0.5
	Pp	30.3 ± 5.4	57.3 ± 11.4	12.3 ± 8.8	0.5 ± 0.9	0

**Table 6:** Sensory assessment and sensory description of samples from variations J (jute bag without pre-drying, without turning), Jp (jute bag with pre-drying), JpT (jute bag with pre-drying and high drying temperature), JpsT (jute bag with pre-drying, shorter fermentation time and higher drying temperature), Jpt (jute bag with pre-drying and turning), P (plastic bag without pre-drying nor turning), Pp (plastic bag with pre-drying), and Wt (wooden box with turning) at locations A and E, *n* = 2. The ratings refer to a 10-cm/10-point scale. Values followed by the same superscript letter within row of each description are not significantly different (*p* < 0.001).

Location	Variations	Acidity	Bitterness	Fruity, Citrus	Fruity, Banana	Floral	Nutty	Woody	Spicy	Cocoa	Roasted-Burnt	Earthy	Astringency
		<0.001	<0.001	<0.001	0.007	0.013	0.272	<0.001	0.015	0.001	<0.001	<0.001	<0.001
A	Wt	7.3 ^ab^	4.6 ^cde^	4.3 ^a^	1.1 ^a^	2.4 ^a^	3.2	2.1 ^cd^	3.1 ^a^	2.4 ^ab^	4.0 ^ab^	1.2 ^c^	4.4 ^bcde^
	J	5.7 ^bc^	4.3 ^cde^	3.6 ^ab^	1.4 ^a^	2.2 ^a^	3	2.2 ^cd^	2.6 ^a^	2.8 ^ab^	4.6 ^ab^	1.4 ^bc^	5.1 ^bcde^
	Jp	1.6 ^d^	5.9 ^abc^	0.6 ^d^	0.9 ^a^	2.2 ^a^	4.2	4.4 ^ab^	1.7 ^a^	3.3 ^ab^	4.5 ^ab^	2.5 ^abc^	5.8 ^abc^
	Jpt	2.5 ^d^	5.8 ^abcd^	1.0 ^cd^	0.9 ^a^	2.8 ^a^	3.9	3.4 ^bc^	1.6 ^a^	3.7 ^a^	4.0 ^ab^	2.2 ^abc^	5.5 ^abcd^
	JpT	2.1 ^d^	6.6 ^ab^	0.7 ^d^	1.4 ^a^	2.3 ^a^	4.8	5.1 ^ab^	1.8 ^a^	2.4 ^ab^	5.0 ^a^	3.1 ^ab^	6.1 ^ab^
	JpsT	1.9 ^d^	7.1 ^a^	0.6 ^d^	1.1 ^a^	2.6 ^a^	4.6	5.4 ^a^	1.6 ^a^	2.1 ^b^	4.9 ^a^	3.8 ^a^	7.1 ^a^
E	Wt	7.4 ^a^	4.0 ^de^	4.6 ^a^	1.5 ^a^	3.5 ^a^	3.6	1.2 ^d^	2.9 ^a^	2.4 ^ab^	3.0 ^b^	1.2 ^c^	3.5 ^e^
	Jp	6.1 ^ab^	3.6 ^e^	2.8 ^abc^	1.3 ^a^	2.7 ^a^	3.4	1.9 ^cd^	2.3 ^a^	2.9 ^ab^	3.4 ^ab^	1.4 ^bc^	3.8 ^de^
	Jpt	6.3 ^ab^	4.8 ^bcde^	3.7 ^ab^	2.0 ^a^	3.5 ^a^	3.6	1.6 ^cd^	2.9 ^a^	2.1 ^b^	2.7 ^b^	1.3 ^c^	4.3 ^cde^
	P	4.3 ^c^	5.3 ^abcde^	2.3 ^bd^	2.4 ^a^	4.0 ^a^	3.6	3.4 ^bc^	2.3 ^a^	3.3 ^ab^	3.7 ^ab^	1.9 ^bc^	5.0 ^bcde^
	Pp	7.0 ^ab^	3.8 ^e^	4.6 ^a^	2.4 ^a^	3.7 ^a^	3.9	1.9 ^cd^	2.3 ^a^	2.7 ^ab^	2.8 ^b^	1.0 ^c^	3.7 ^e^

Mixed ANOVA with product as fixed variable (*p*-values displayed) and assessor as random variable. Mean value of samples and significance levels were given in case of significant product effect in ANOVA.

## Data Availability

The data presented in this study are available in article and supplementary material.

## Supplementary Materials

The following supporting information can be downloaded at: https://www.mdpi.com/article/10.3390/foods13010137/s1, Figure S1: Time until reaching maximum temperature Tmax (h) during the fermentations at location A in red and at location E in blue for the following variations: J (jute bag without pre-drying, without turning; location A: n = 3, E: n = 4)/P (plastic bag without pre-drying nor turning; E: n = 2), Jp (jute bag with pre-drying; E: n = 6)/JpT (jute bag with pre-drying and high drying temperature; A: n = 3)/Jpt (jute bag with pre-drying and turning; A: n = 3, E: n = 5)/Pp (plastic bag with pre-drying; E: n = 4)/PpT (plastic bag with pre-drying and high drying temperature; A: n = 3).
