# Supplementary material for: Variations in Ecuadorian Cocoa Fermentation and Drying at Two Locations: Implications for Quality and Sensory
Source: Foods. 2023 Dec 30;13(1):137. doi: 10.3390/foods13010137 (PMC10778537; doi:10.3390/foods13010137)
Supplement: Supplementary file 1 [file foods-13-00137-s001.zip › foods-2777892-supplementary.pdf]

Supplementary Material

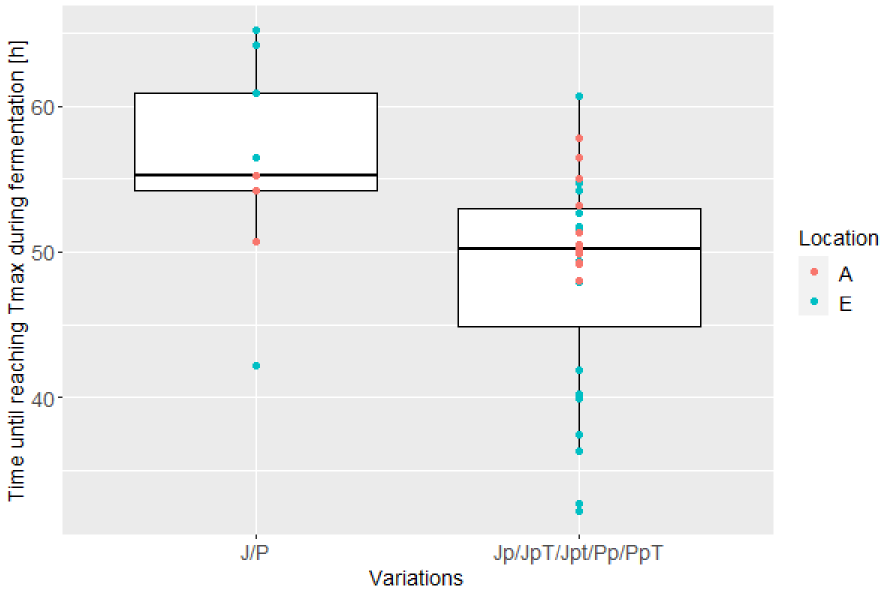

Figure S1. Time until reaching maximum temperature Tmax (h) during the fermentations at location A in red and at location E in blue for the following variations: J (jute bag without pre-drying, without turning; location A: $n = 3$, E: $n = 4$)/P (plastic bag without pre-drying nor turning; E: $n = 2$), Jp (jute bag with pre-drying; E: $n = 6$)/JpT (jute bag with pre-drying and high drying temperature; A: $n = 3$)/Jpt (jute bag with pre-drying and turning; A: $n = 3$, E: $n = 5$)/Pp (plastic bag with pre-drying; E: $n = 4$)/PpT (plastic bag with pre-drying and high drying temperature; A: $n = 3$).
